# Complexation of multiple mineral elements by fermentation and its application in laying hens

**DOI:** 10.3389/fnut.2022.1001412

**Published:** 2022-09-29

**Authors:** Huayou Chen, Xinyu Heng, Keyi Li, Zhen Wang, Zhong Ni, Ebin Gao, Yangchun Yong, Xin Wu

**Affiliations:** ^1^School of Life Sciences, Jiangsu University, Zhenjiang, China; ^2^State Key Laboratory of Biochemical Engineering, Institute of Process Engineering, Chinese Academy of Sciences, Beijing, China; ^3^Key Laboratory of Agro-ecological Processes in Subtropical Region, Institute of Subtropical Agriculture, Chinese Academy of Sciences, Changsha, China

**Keywords:** mineral element complex, laying hen, synergistic fermentation, bean dregs, soybean meal, mineral element absorption

## Abstract

To overcome the problems with current mineral supplements for laying hens including low absorption, mineral antagonism, and high cost, we developed mineral element fermentation complexes (MEFC) by synergistically fermenting bean dregs and soybean meal with strains and proteases and complexing with mineral elements. The fermentation complexation process was optimized based on the small peptide and organic acid contents and the complexation rate of mineral elements after fermentation. The optimal conditions were as follows: the total inoculum size was 5% (v/w), 15% (w/w) wheat flour middling was added to the medium, and mineral elements (with 4% CaCO_3_) were added after the completion of aerobic fermentation, fermentation at 34°C and 11 days of fermentation. Under these conditions, the complexation rates of Ca, Fe, Cu, Mn, and Zn were 90.62, 97.24, 73.33, 94.64, and 95.93%, respectively. The small peptide, free amino acid, and organic acid contents were 41.62%, 48.09 and 183.53 mg/g, respectively. After 60 days of fermentation, 82.11% of the Fe in the MEFC was ferrous ions, indicating that fermentation had a good antioxidant effect on ferrous ion, and the antioxidant protection period was at least 60 days. Fourier transform infrared spectroscopy showed that the mineral ions were complexed with amino and carboxyl groups. The added mineral elements promoted microbial growth, protein degradation, and organic acid secretion and significantly improved fermentation efficiency. Animal experiments showed that MEFC had positive effects on several parameters, including production performance (average daily feed intake, *P* < 0.05; egg production rate, *P* < 0.05; and average egg weight, *P* < 0.05), mineral absorption, intestinal morphology (villus height to crypt depth ratio in the jejunum and ileum, *P* < 0.05), and blood routine and biochemical indexes (red blood cells, *P* < 0.05; hemoglobin, *P* < 0.05). This study provides theoretical support for the development of mineral complexes for laying hens via fermentation.

## Introduction

Mineral elements are essential for maintaining the normal physiological functions and health of all organisms and greatly impact the production performance of laying hens (1, 2). Ca, Fe, Cu, Zn, and Mn are essential elements in laying hens, and deficiency of these minerals has been reported to cause growth stagnation, impaired immunity, decreased egg production rates, and other health problems (3, 4). During breeding, laying hens are often fed large amounts of inorganic mineral salts and stone powder. However, these mineral salts cannot be fully absorbed, leading to high levels of excretion and the potential for environmental pollution. In addition, excessive intake of inorganic mineral salts can lead to heavy metal poisoning, which causes serious harm to laying hens. Absorption of Ca^2+^ from stone powder requires degradation in the digestive tract to release free Ca^2+^, resulting in a low rate of calcium absorption.

The mineral supplements commonly administered to laying hens, such as inorganic mineral salts, organic acid mineral salts, amino acid chelates, and small peptide complexes, have several disadvantages. Inorganic mineral salts, which are widely used because of their low cost, have a low absorption rate, poor stability, and absorption antagonism with other minerals (3, 5). Organic acid mineral salts are absorbed at a higher rate than inorganic mineral salts but are hampered by precipitation and absorption competition (6). Although amino acid chelates have a high absorption rate, strong stability, and safety, they are expensive (7). Small peptide complexes are better than the others, but few commercial products exist. Hence, developing appropriate mineral supplements for the poultry industry is vital. The absorption pathways of each type of mineral element will be saturated, and supplementing a single type of mineral element may not be the best choice. By supplementing multiple types of mineral elements simultaneously, the various ways of absorbing mineral elements can be fully utilized to achieve the best absorption. Therefore, developing mineral element complexes containing multiple types of mineral elements has great potential for applications and research.

Bean dregs (BD) and soybean meal (SBM) are rich in proteins and sugars (8), which are ideal sources of mineral complexing ligands. Fermentation is an efficient, low-cost method for degrading proteins, starch, and other macromolecular substances to produce amino acids, small peptides, organic acids, and other complexing ligands (9). Heng et al. (10) reported that cooperative fermentation by *Bacillus*, yeast, and lactic acid bacteria with proteases greatly improved fermentation efficiency. By combining BD and SBM fermentation with mineral complexation, fermented mineral complexes can be obtained via a simple, highly efficient production process. The obtained mineral element fermentation complexes (MEFC) contain various types of mineral elements, such as small peptide complexes, amino acid complexes, and organic acid complexes.

In this study, we used *Bacillus subtilis*, *Saccharomyces cerevisiae*, *Lactobacillus plantarum*, *Lactobacillus rhamnosus*, acid protease, and papain to synergistically ferment BD and SBM and then optimized the mineral element fermentation complexation process. The effect of the added mineral elements on fermentation was investigated by analyzing the produced MEFC. The effects of feeding MEFC on laying hens were analyzed in animal experiments. The results of this study provide a feasible strategy for producing mineral element complexes at low cost with high efficiency, which contributes to the supply of mineral elements for laying hens.

## Materials and methods

### Materials

BD and SBM were purchased from a local bean processing factory. All solvents and chemicals were of analytical grade or better. Both acid protease activity and papain activity were 60,000 u/g. *L. plantarum* (CGMCC 1.557) and *L. rhamnose* (CGMCC 1.2467) were purchased from China General Microbiological Culture Collection Center. The *B. subtilis* and *S. cerevisiae* strains used were screened and preserved in our laboratory.

### Cultivation of fermentation strains

*Bacillus subtilis*, *L. plantarum*, and *L. rhamnose* were cultured in soymilk medium (2% [w/v] soybeans, 2% [w/v] brown sugar) at 37°C for 18 h. *S. cerevisiae* was cultured in soymilk medium at 28°C for 18 h.

### Initial solid-state fermentation

The fermentation medium contained 100 g of BD and 100 g of SBM. First, the medium was inoculated with *B. subtilis* for aerobic fermentation at 30°C for 12 h. Next, *S. cerevisiae*, *L. plantarum*, *L. rhamnosus*, 0.65% acid protease, and 0.65% papain were added to the medium, which was placed in a sealed fermentation bag containing a one-way vent for anaerobic fermentation and incubated at 30°C for 7 days. An equal volume of each strain was inoculated, and the total inoculum size was 5% (v/w).

### Optimization of fermentation complexation

According to the 5% MEFC addition amount and standard mineral elements required for laying hens, 796 mg of FeSO_4_⋅7H_2_O, 32 mg of CuSO_4_⋅5H_2_O, 492 of mg MnSO_4_⋅H_2_O, 528 mg of ZnSO_4_⋅7H_2_O, and 4% CaCO_3_ were added per each 100 g of fermented bean dregs and soybean meal (FBDSM). To determine the optimal timing for adding the mineral elements, mineral elements were added after the completion of aerobic fermentation and after the completion of anaerobic fermentation. The complexing times of the former were the same as the anaerobic fermentation time. The complexing times of the latter were 0.5, 1, 3, 6, 12, and 24 h. To determine the optimal parameters for mineral complexation rates, other conditions were varied as follows: CaCO_3_: 0, 2, 4, 6, 8, 10, 12, 14, and 16% (w/w); carbon source: wheat bran, wheat flour middlings, wheat flour, rice bran, corn flour, and vinegar residue [all at 15% (w/w)]; fermentation temperature: 22°C, 25°C, 28°C, 31°C, 34°C, 37°C, and 40°C; inoculum size: 0.5, 1, 3, 5, 7, and 9%, (v/w); and fermentation time: 1, 3, 5, 7, 9, 11, 13, 15, and 20 days. Furthermore, the small peptide and organic acid contents were evaluated during optimization.

### Fourier transform infrared spectroscopy analysis

Fourier transform infrared spectroscopy (FTIR) analysis was performed according to the method described by Hou et al. (11), with some modifications. MEFC (1 g) was washed three times with 30 ml of anhydrous ethanol, dried at 45°C, ground, and then analyzed using a Fourier transform infrared spectrometer (Nicolet iS50; USA).

### Effect of fermentation on the redox of Fe^2+^ and Fe^3+^

FeSO_4_⋅7H_2_O and FeCl_3_ containing equal amounts of Fe were added to the fermentation medium, respectively, to investigate the redox changes of Fe^2+^ and Fe^3+^.

### Effect of adding minerals on fermentation

Using a fermentation without added mineral elements as the control, the corresponding indicators were analyzed to evaluate the effect of mineral elements on fermentation. Samples were taken at 11 days to analyze protein degradation and free amino acids and organic acids. The number of live fermenting microorganisms was determined at 0, 0.5, 1, 4, 7, 11, 15, 20, and 30 days. Quantitative real-time PCR (qPCR) of *B. subtilis* was performed at 0, 0.5, 1, 4, 7, 11, 15, 20, and 30 days, and qPCR of *S. cerevisiae*, *L. plantarum*, and *L. rhamnosus* was performed at 0.5, 1, 4, 7, 11, 15, 20, and 30 days.

### Determination of the mineral element complexation rate

The mineral element complexation rate was determined based on Chinese national standard GB 5009.268–2016 (12), with appropriate modifications. The added mineral element content was recorded as Fe_1_, Cu_1_, Mn_1_, Zn_1_, and Ca_1_. MEFC (1.00 g) was mixed with 30 ml of anhydrous ethanol, shaken, soaked for 1 h, and then centrifuged at 6,000 × *g* for 15 min. Subsequently, 10 ml of the supernatant was ashed in a muffle furnace. The Fe, Cu, Mn, and Zn contents were measured by inductively coupled plasma-optical emission spectroscopy (ICP-OES) (VARIAN VISTA-MPX; USA) and was recorded as Fe_2_, Cu_2_, Mn_2_, and Zn_2_, respectively. FBDSM was used as a control and was recorded as Fe_3_, Cu_3_, Mn_3_, and Zn_3_ and was determined by the same method as MEFC.

MEFC (1.00 g) was mixed with 100 ml of 1% CH_3_COOH, shaken, soaked for 1 h, and centrifuged at 6,000 × *g* for 15 min. Then, the supernatant (10 ml) was collected and ashed in a muffle furnace. Ca content was measured using ICP-OES and reported as Ca_2_. MEFC (2.00 g) was mixed with 100 ml of deionized water, shaken, soaked for 1 h, and then centrifuged at 6,000 × *g* for 15 min. The supernatant (10 ml) was ashed in a muffle furnace. Ca content was measured using ICP-OES and was reported as Ca_3_. The following formulae were used to calculate the complexation rates of each mineral element:


(1) $$\text{Fe complexation rate (\%) = }\frac{{\text{(Fe}}_{1}-{\text{Fe}}_{2}+{\text{Fe}}_{3})}{{\text{Fe}}_{1}}\times 100$$

(2) $$\text{Cu complexation rate (\%) = }\frac{{\text{(Cu}}_{1}-{\text{Cu}}_{2}+{\text{Cu}}_{3})}{{\text{Cu}}_{1}}\times 100$$

(3) $$\text{Mn complexation rate (\%) = }\frac{{\text{(Mn}}_{1}-{\text{Mn}}_{2}+{\text{Mn}}_{3})}{{\text{Mn}}_{1}}\times 100$$

(4) $$\text{Zn complexation rate (\%) = }\frac{{\text{(Zn}}_{1}-{\text{Zn}}_{2}+{\text{Zn}}_{3})}{{\text{Zn}}_{1}}\times 100$$

(5) $$\text{Ca complexation rate (\%) = }\frac{{\text{(Ca}}_{1}-{\text{Ca}}_{2}+{\text{Ca}}_{3})}{{\text{Ca}}_{1}}\times 100$$

### Determination of small peptide, free amino acid, and organic acid contents

Small peptide content (including small peptides and free amino acids) was determined as previously described by Shi et al. (13). Dried MEFC (3.00 g) was suspended in 50 ml of 15% trichloroacetic acid, incubated for 10 min, filtered with qualitative filter paper, and centrifuged at 2,600 × *g* for 10 min. The supernatant (10 ml) was used for the determination.

Free amino acids were determined using the method described by Heng et al. (10). The total free amino acid content was calculated as the sum of all free amino acids.

Organic acids were determined using the method described by Moghaddam et al. (14), with slight modifications. The organic acid content includes organic acid group anions and organic acid molecules. The total organic acid content was calculated as the sum of each acid content. MEFC (2.00 g) was mixed with 30 ml of HCl solution (pH 2.7), sonicated for 30 min, shaken, incubated for 3 h, and then centrifuged at 5,500 × *g* for 30 min. The supernatant was diluted with HCl solution (pH 2.0) at a 1:1 ratio, and then filtered through a 0.45 μm filter before high-performance liquid chromatography (HPLC) analysis (SHIMADZU, Japan). The chromatographic conditions were as follows: mobile phase, 0.01 mol/L KH_2_PO_4_ (pH 2.7):methanol, 97:3; chromatographic column, Welch AQ-C18; detection wavelength, 210 nm; and flow rate, 0.6 ml/min.

### Sodium dodecyl sulfate-polyacrylamide gel electrophoresis

SDS-PAGE was performed as previously described by Shi et al. (13). The test samples were dried at 105°C, and then 1 g of the dried sample was mixed with 30 ml of Tris-HCl (0.03 mol/L, pH 8.0), incubated for 1 h, and centrifuged at 6,520 × *g* for 10 min. The supernatant was used for SDS-PAGE.

### Determination of Fe^2+^ content

Fe^2+^ was determined according to the method of Stookey (15), with appropriate modifications. Each sample (2.00 g) was mixed with 600 ml of 1% Cl_3_CCOOH, sonicated for 20 min, and filtered through qualitative filter paper. A 1 ml sample of the filtrate was mixed with 1 ml of acetate buffer (pH 6.0) containing 4.0 mmol/L phenazine and 0.1 mol/L Na_2_S_2_O_3_. After incubation at 26°C in a water bath for 35 min, the mixture was centrifuged at 6,000 × *g* for 15 min. The absorbance of the supernatant was measured at 562 nm. Each sample (1.00 g) was completely ashed in a muffle furnace and then the total Fe content was determined by ICP-OES. The Fe^2+^ content was calculated as follows:


(6) $${\text{Fe}}^{2+}content \% =\frac{{\text{Fe}}^{2+}}{\text{Total Fe}}\times 100$$

### Determination of fermenting microorganisms

The number of live fermenting microorganisms was determined according to ISO:4833:2003 (16). A sample (1.00 g) of the fermentation was mixed with 10 ml of sterile water and incubated for 30 min, and then the liquid was diluted and spread on solid medium for culturing.

DNA extraction and qPCR of fermenting microorganisms were performed according to the method described by Yu et al. (17). Each sample (1.00 g) was mixed with 10 ml of deionized water, shaken for 20 min, and filtered through a 200-mesh sieve. A sample of the filtrate (2 ml) was used to extract DNA using the FastPure Bacteria DNA Isolation Mini Kit (Vazyme, China) for qPCR (QuantStudio 3 Real-Time PCR System; Applied Biosystems, USA). The specific primers used for the strains (18, 19) are listed in Table 1.

**TABLE 1:** Specific primers of strains.

Strains	Primers (5′ - 3′)
*Bacillus subtilis*	F: CGTAGAGCCACTTGAGCG R: CTGCCGTTACAGTTCCTT
*Saccharomyces cerevisiae*	F: GCGATAACGAACGAGACCCTAA R: CCAGCACGACGGAGTTTCACAAGAT
*Lactobacillus plantarum*	F: GTGGTGCGGTCGATATTTTAGTT R: TCAGCCGCGCTTGTAACC
*Lactobacillus rhamnosus*	F: GACGCAGCCGGTTGACCCAA R: GGCGGCAGTTGCCCCAGAAT

### Animal experiments

#### Animal ethics

All experimental animal procedures were reviewed and approved by the Institutional Animal Care and Use Committee of Jiangsu University (UJS IACUC) (approval number: UJS-IACUC-2020041001) and were performed in accordance with the National Institutes of Health Guide for the Care and Use of Laboratory Animals.

#### Experimental design and feeding management

Seventy-twoSan-Huang laying hens with the same body conditions and similar body weights were selected for the experiment. The hens were divided into experimental and control groups, with three replicates in each group and 12 laying hens in each replicate. The feed for laying hens was prepared based on the “Nutrient Requirements of Poultry” of the NRC (20), and the composition is shown in Table 2. The feed for the experimental group contained 5% MEFC, and the feed for the control group contained 5% unfermented MEFC raw material containing the same amounts of FeSO_4_⋅7H_2_O, CuSO_4_⋅5H_2_O, MnSO_4_⋅H_2_O, ZnSO_4_⋅7H_2_O, and CaCO_3_ as in the MEFC. Corn, multivitamin, DL-methionine, stone, CaHPO_4_, vitamin C, and phytase were all feed grade.

**TABLE 2:** Feed formula for laying hens (as-fed basis, %).

Item	Experimental group	Control group
**Ingredients**		
Corn	62	62
Soybean meal	26.28	26.28
Multivitamin	0.05	0.05
DL-methionine	0.12	0.12
Stone	5	5
CaHPO_4_	1.4	1.4
Vitamin C	0.1	0.1
Phytase	0.05	0.05
Mineral element	5 MEFC	5 unfermented MEFC raw material
**Nutrient content**		
AME, MJ/kg	11.76	11.58
Crude protein	18.12	17.97
Available phosphorus	0.40	0.39
Methionine	0.41	0.41
Calcium	2.39	2.39
Fe	0.016	0.016
Cu	0.0012	0.0012
Mn	0.0093	0.0093
Zn	0.0086	0.0086

5% unfermented MEFC raw material contained the same amount of FeSO_4_7H_2_O, CuSO_4_5H_2_O, MnSO_4_H_2_O, ZnSO_4_7H_2_O and CaCO_3_ as MEFC.

The pre-experiment period lasted for 15 days, and the formal experiment lasted for 60 days. Laying hens were exposed to light for 16 h per day and were fed twice a day at 8:30 and 17:30. The laying hens were given free access to food and water. The laying hen house was disinfected once every 7 days, and the feces were cleared at 15:00 every day. Eggs were collected every afternoon before feeding. Other management measures were performed according to the routine feeding management of laying hens. The experiment was repeated thrice.

#### Determination of production performance

The amount of feed, remaining feed, number of eggs, egg weight, number of broken soft eggs, and eggshell thickness were recorded daily. Average daily feed intake, egg production rate, average egg weight, broken soft egg rate, and average eggshell thickness were calculated every 30 days using the following formulae:


(7) Average daily feed intake (g)

$$=\frac{\text{Total feed amount - Total remaining feed amount}}{\text{Total number of laying hens }\times \text{ 30}}$$

(8) Egg production rate (%)

$$=\frac{\text{Total number of eggs}}{\text{Total number of laying hens }\times \text{ 30}}\times 100$$

(9) Average eggweight (g)

$$=\frac{\text{Total eggweight}}{\text{Total number of eggs }}\times 100$$

(10) Broken soft eggrate (%)

$$=\frac{\text{Total number of broken soft eggs}}{\text{Total number of eggs }}\times 100$$

(11) Average eggshell thickness (mm)

$$=\frac{\text{Total eggshell thickness}}{\text{Total number of eggs }}\times 100$$

#### Determination of the mineral element contents in the blood, liver, heart, and feces

Before feeding on the morning of the 61st day, feces were collected from each replicate to measure the mineral contents, and the average value was calculated. The laying hens were slaughtered after blood sampling, and the blood, liver, and heart were collected to determine the mineral element content. Mineral element content was determined according to the Chinese national standard GB 5009.268-2016 (12).

#### Preparation of intestinal paraffin sections

On the 61st day, the hens were slaughtered after blood sampling, and the duodenum, jejunum, and ileum were collected and soaked in 10% neutral formalin overnight. Intestinal paraffin sections were prepared according to the method of Wang et al. (21). For each intestinal section, the average of five measured values was calculated.

#### Determination of routine blood and biochemical parameters

After a 12 h fast on the 61st day, 5 ml of blood was collected from the wing fossa vein of each laying hen, incubated in a refrigerator at 4°C for 30 min, and then centrifuged at 1,800 × *g* for 15 min. Routine blood tests were performed using an automatic animal blood cell analyzer (BC-2800Vet; Mindray, China), including white blood cells (WBC), red blood cells (RBC), hemoglobin, and hematocrit. Biochemical indexes were determined using an automatic biochemical analyzer (AU680; Beckman Coulter, USA), including alanine transaminase (ALT), aspartate transaminase (AST), urea, uric acid, blood glucose, total cholesterol (TCHOL), high-density cholesterol (HDL-C), and low-density cholesterol (LDL-C).

### Statistical analysis

All experiments were performed in triplicate. All data are expressed as the mean ± SD and were analyzed using one-way analysis of variance (ANOVA) in SPSS and plotted using Origin Pro8. Statistical significance was set at *P* < 0.05.

## Results and discussion

### Effect of the timing of mineral element addition on the complexation rate

**FIGURE 1:**
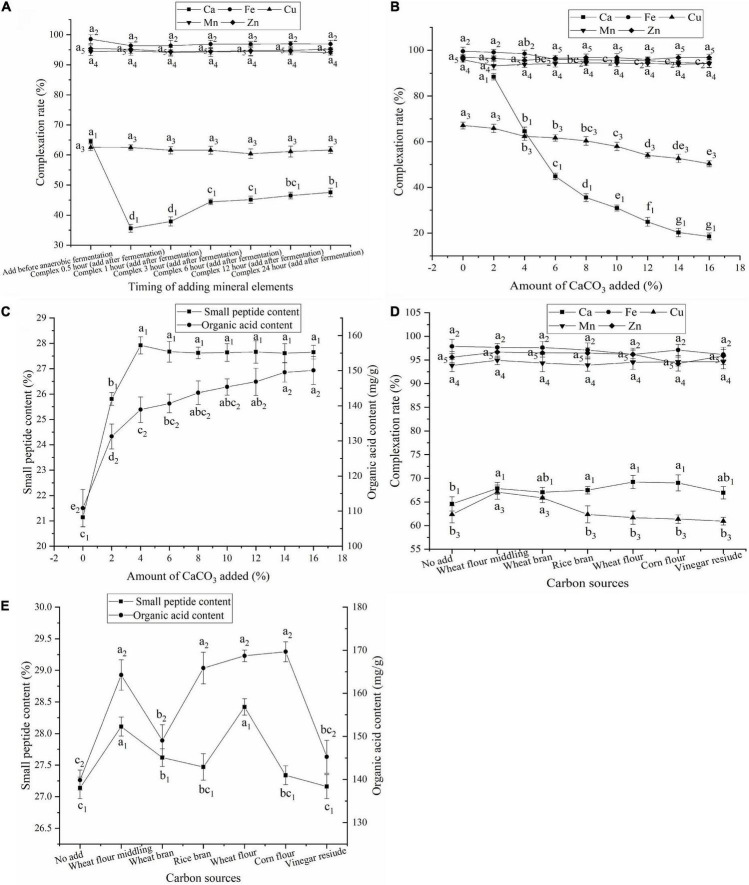
**(A)** Effect of timing of adding mineral elements on the complexation rate; **(B)** Effect of amount of CaCO_3_ added on the complexation rate; **(C)** Effect of amount of CaCO_3_ added on contents of small peptides and organic acids; **(D)** Effect of carbon sources on the complexation rate; **(E)** Effect of carbon sources on contents of small peptides and organic acids; Values expressed as mean ± standard deviation (*n* = 3).

As shown in Figure 1A, the complexation rates of Fe, Cu, Mn, and Zn were similar regardless of whether mineral elements were added after aerobic fermentation or after anaerobic fermentation. This may be because of the good solubility of FeSO_4_⋅7H_2_O, CuSO_4_⋅5H_2_O, MnSO_4_⋅H_2_O, and ZnSO_4_⋅7H_2_O, which can dissociate quickly in the fermentation medium to release free Fe^2+^, Cu^2+^, Mn^2+^, and Zn^2+^. Complexation of free Fe^2+^, Cu^2+^, Mn^2+^, and Zn^2+^ was completed in a short time, and the analysis showed a high complexation rate within 0.5 h. The complexation rate of Cu was much lower than the rates of Fe, Mn, and Zn, which may be because Cu did not easily form a complex under these conditions. There is no difference if Fe, Cu, Mn and Zn are added after completion of aerobic or anaerobic fermentation. Therefore, to reduce the process steps and production costs, Fe, Cu, Mn, and Zn were added after aerobic fermentation.

The Ca complexation rate was significantly higher when CaCO_3_ was added after aerobic fermentation than after anaerobic fermentation (Figure 1A). Ca complexation can only occur after CaCO_3_ is degraded by organic acids to release free Ca^2+^. The CaCO_3_ added after anaerobic fermentation could not be completely degraded to free Ca^2+^ by organic acids due to the short fermentation time, which affected Ca complexation. Therefore, CaCO_3_ was added after aerobic fermentation.

### Effect of the amount of CaCO_3_ added

The effect of CaCO_3_ addition on the complexation rate of mineral elements was investigated, and the results are shown in Figure 1B. The complexation rates of Ca and Cu decreased with increasing amounts of CaCO_3_. However, the addition of CaCO_3_ had no significant effects on the complexation rates of Fe, Mn, and Zn. An increase in CaCO_3_ increased the amount of Ca^2+^ released. However, when it exceeded the degradation limit of the organic acids in FBDSM, the amount of Ca^2+^ released remained unchanged. Thus, the Ca complexation rate decreased as the amount of CaCO_3_ increased. The decrease in the Cu complexation rate may be due to ligand competition caused by Ca complexation.

The small peptide content of FBDSM with no mineral elements was 24.59%, and the organic acid content was 120.63 mg/g (Supplementary Table 1), while the small peptide content of FBDSM with Fe, Cu, Mn, and Zn was 21.14% and the organic acid content was 110.86 mg/g (Figure 1C). This indicated that the addition of Fe, Cu, Mn, and Zn may affect the growth and metabolism of the fermenting microorganisms and decrease the small peptide and organic acid contents. The addition of the four minerals created a high salt environment, which affected the osmotic pressure on the cell membrane of the fermenting microorganisms, slowing their metabolism and even causing death. In addition, Cu has a strong bactericidal effect (22) and a great impact on microorganisms.

The small peptide content increased as the amount of CaCO_3_ added increased and reached a maximum of 27.92% at 4% CaCO_3_ (Figure 1C). The organic acid content increased continuously following the addition of CaCO_3_ (Figure 1C). Fan et al. (23) found that Ca^2+^ increased protease activity, which could promote protein degradation. CaCO_3_ neutralizes organic acids, slows microbial inhibition caused by low pH, and promotes the secretion of organic acids by microorganisms. When a higher amount of CaCO_3_ was added, the undegraded CaCO_3_ was very stable and had less influence on fermentation and the complexation of the other mineral elements, but led to CaCO_3_ waste. When 4% CaCO_3_ was added, the Ca and Cu complexation rates were greater than 60%; the Fe, Mn, and Zn complexation rates were greater than 90%; and the small peptide and organic acid contents were 27.92% and 138.92 mg/g, respectively. The fermentation complexation effect satisfied the requirements. Therefore, 4% CaCO_3_ was added.

### Effect of carbon source

Carbon source is essential for microbial growth and provides precursors for the production of organic acids (24). The organic acid content in the fermentation medium is closely related to the amount of free Ca^2+^ released by acidolysis of CaCO_3_ and the Ca complexation rate. As shown in Figure 1D, changing the carbon source did not have a significant effect on the complexation rates of Fe, Mn, and Zn but did have an effect on the complexation rates of Ca and Cu. As shown in Figures 1D,E, when corn flour was added, the organic acid content was the highest, but the small peptide content was lower, resulting in fewer complexing ligands and a lower Ca complexation rate. When wheat flour was added, the Ca complexation rate and small peptide content were highest; however, the Cu complexation rate was lower. When wheat flour middling was added, the complexation rate, organic acid content, and small peptide content were not the highest, but all values were in the middle to upper range, and the Cu complexation rate was the highest. Therefore, wheat flour middling was used.

### Effect of fermentation temperature

**FIGURE 2:**
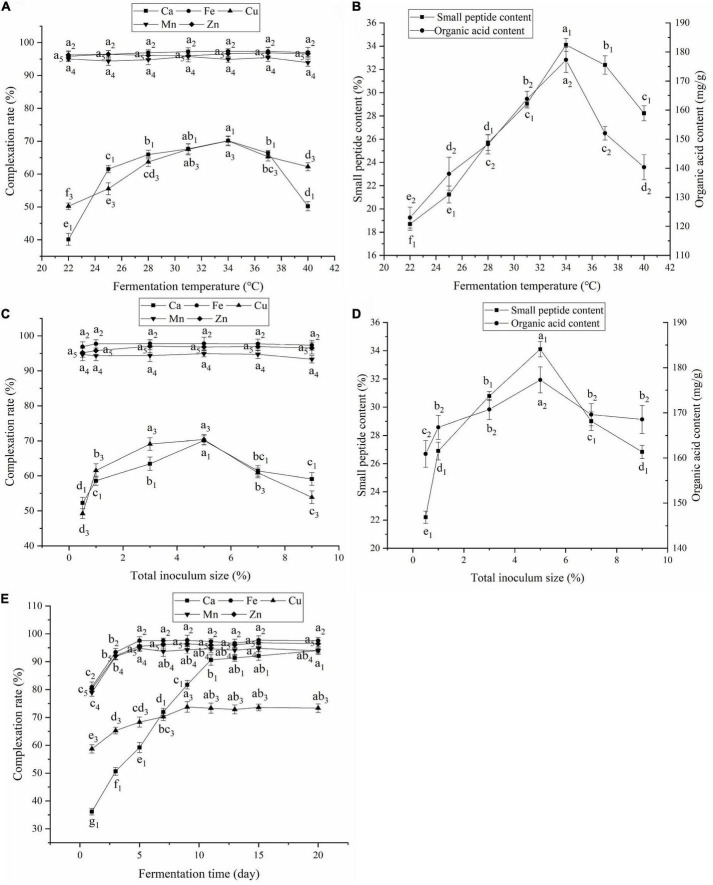
**(A)** Effect of fermentation temperature on the complexation rate; **(B)** Effect of fermentation temperature on contents of small peptides and organic acids; **(C)** Effect of total inoculum size on the complexation rate; **(D)** Effect of total inoculum size on contents of small peptides and organic acids; **(E)** Effect of fermentation time on the complexation rate. Values expressed as mean ± standard deviation (*n* = 3).

The fermentation temperature is closely related to complexation, enzymatic hydrolysis, and microbial fermentation. As shown in Figures 2A,B, changing the fermentation temperature had almost no effect on the complexation rates of Fe, Mn, and Zn. However, as the temperature increased, the complexation rates of Ca and Cu and the small peptide and organic acid contents increased and then decreased, reaching their highest values at 34°C. Low or high temperatures were not conducive to the reproduction and metabolism of the fermentation strains and enzyme activity (25), resulting in fewer complexing ligands, such as small peptides and organic acids, and affecting the complexation of Ca and Cu. Therefore, 34°C was selected as the fermentation temperature.

### Effect of inoculum size

As shown in Figures 2C,D, inoculum size had little effect on the complexation rates of Fe, Mn, and Zn. However, as inoculum size increased, the complexation rates of Ca and Cu, small peptide content, and organic acid content first increased and then decreased, peaking at 5% inoculum. When the inoculum size was small, fermentation was slow, the organic acid and small peptide contents were low, and the complexation rates of Ca and Cu were low. When the inoculum size was increased, the fermentation strains multiplied rapidly, accelerating fermentation and CaCO_3_ degradation. However, too much inoculum could cause more organic acids to be secreted prematurely, inhibiting strain growth and protease activity (26) and reducing small peptide and organic acid contents. Additionally, microorganisms have an enriching effect on mineral elements. As inoculum size increased, Ca and Cu were enriched by microorganisms, which led to a reduction in the complexation rate. Therefore, the inoculum size was set at 5%.

### Effect of fermentation time on the complexation rate

As shown in Figure 2E, as the fermentation time increased, the complexation rates of Fe, Cu, Mn, Zn, and Ca increased. The Fe, Mn, and Zn complexation rates leveled off after 5 days, and the overall complexation rate was higher. The Cu complexation rate leveled off after 9 days, and the Ca complexation rate leveled off after 11 days. Extended fermentation may lead to contamination with harmful bacterial and increased fermentation costs. Therefore, the fermentation time was set to 11 days.

### Fourier transform infrared spectroscopy analysis

**FIGURE 3:**
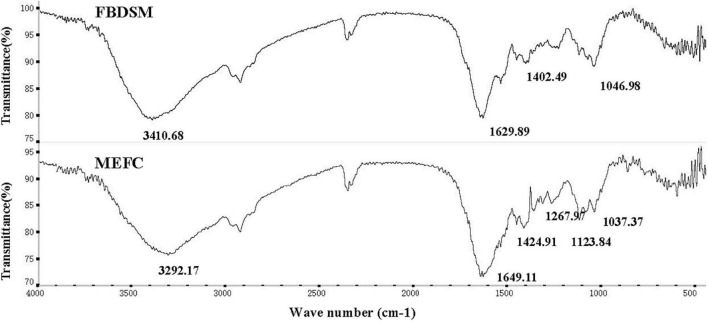
Fourier transform infrared spectroscopy analysis. FBDSM: fermented bean dregs and soybean meal; MEFC: mineral element fermentation complexes.

Studies have shown that mineral ions can be complexed with the amino and carboxyl groups of small molecules, such as peptides, amino acids, and organic acids, as well as nitrogen and oxygen atoms containing lone pairs of electrons in the side chains (27). As shown in Figure 3, the peak generated by the stretching vibration of N-H at 3,410.68 cm^–1^ shifted to 3,292.17 cm^–1^ in MEFC, and the peak shape became wider, indicating that mineral ions were complexed with -NH_2_. The symmetrical vibration of -COOH is generally around 1,400 cm^–1^, and the anti-symmetric vibration is around 1,650 cm^–1^. The peak at 1,402.49 cm^–1^ in the map of FBDSM without mineral elements shifted to 1,424.91 cm^–1^ in the high band in the MEFC map. Simultaneously, the C = O absorption peak at 1,629.89 cm^–1^ shifted to 1,649.11 cm^–1^. These results indicated that mineral ions were complexed complex with -COOH.

### Effect of mineral elements on protein degradation

**FIGURE 4:**
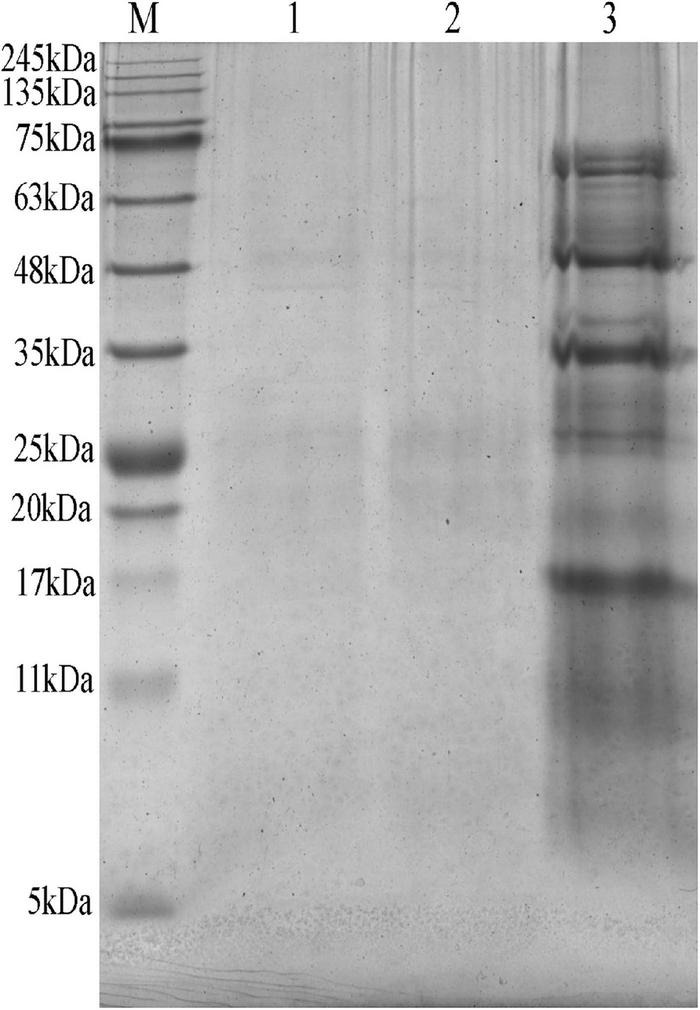
SDA-PAGE of fermented bean dregs and soybean meal (FBDSM) and mineral element fermentation complexes (MEFC) proteins. M: protein molecular weight markers (5∼245kDa); 1: FBDSM; 2: MEFC; 3: raw materials.

As shown in Supplementary Table 2, the small peptide content of MEFC was slightly higher than that of FBDSM, but the difference was not significant. Compared with the raw materials, regardless of whether mineral elements were added or not, proteins greater than 5kDa were almost completely degraded, and the degree of degradation was similar (Figure 4). This indicated that the addition of mineral elements did not affect protein degradation.

### Free amino acid and organic acid analysis

Free amino acids, except arginine, were increased from 40.07 to 48.09 mg/g after adding mineral elements (Table 3). This indicated that the added mineral elements promoted protein degradation and release more amino acids from the raw materials.

**TABLE 3:** Free amino acid analysis.

Free amino acids	FBDSM, mg/g	MEFC, mg/g
Aspartic acid	1.83 ± 0.32^a^	1.9 ± 0.27^a^
Glutamic acid	4.36 ± 1.26^a^	4.96 ± 0.76^a^
Serine	1.54 ± 0.33^b^	2.4 ± 0.65^a^
Arginine	1.96 ± 0.18^a^	1.00 ± 0.16^b^
Glycine	0.62 ± 0.08^b^	1.06 ± 0.14^a^
Threonine	0.92 ± 0.12^b^	1.63 ± 0.25^a^
Proline	1.75 ± 0.28^a^	1.95 ± 0.19^a^
Alanine	1.96 ± 0.69^b^	2.68 ± 0.85^a^
Valine	1.69 ± 0.11^b^	2.27 ± 0.72^a^
Methionine	0.31 ± 0.09^b^	0.4 ± 0.12^a^
Isoleucine	2.43 ± 0.58^a^	2.79 ± 0.23^a^
Leucine	10.09 ± 1.42^b^	12.18 ± 1.14^a^
Histidine	4.21 ± 0.45^b^	5.11 ± 0.23^a^
Lysine	4.42 ± 0.79^a^	4.52 ± 0.92^a^
Tyrosine	1.98 ± 0.61^b^	3.24 ± 0.07^a^
Total free amino acid content	40.07 ± 1.1^b^	48.09 ± 1.52^a^

FBDSM, fermented bean dregs and soybean meal; MEFC, mineral element fermentation complexes. Values expressed as mean ± standard deviation (n = 3).

Means with different letters are significantly different (P < 0.05).

The addition of mineral elements increased the total organic acid content from 158.4 to 183.53 mg/g (Table 4), suggesting that mineral elements may activate related enzymes, promote the metabolism of sugars, and increase the production of organic acids. CaCO_3_ neutralized organic acids and slowed the inhibition of strain metabolism and enzyme activity induced by low pH. Zheng et al. (28) used 17% NaOH as a neutralizer to promote lactic acid production by *Sporolactobacillus inulinus*. Sugar metabolism, including glycolysis and the citric acid cycle, is a dynamic process in which acids are consumed and metabolized to produce other acids (29). The lactic acid content increased remarkably from 74.38 to 94.06 mg/g, and the acetic acid content increased from 27.56 to 36.79 mg/g.

**TABLE 4:** Organic acid analysis.

Organic acids	FBDSM, mg/g	MEFC, mg/g
Tartaric acid	22.73 ± 2.89^a^	20.84 ± 3.11^a^
Formic acid	17.18 ± 1.95^a^	15.08 ± 2.35^b^
Malic acid	5.65 ± 1.2^a^	4.2 ± 1.02^b^
Lactic acid	74.38 ± 3.73^b^	94.06 ± 3.89^a^
Acetic acid	27.56 ± 2.83^b^	36.79 ± 3.36^a^
Citric acid	3.64 ± 0.46^a^	2.23 ± 0.75^b^
Succinic acid	4.15 ± 1.54^b^	6.59 ± 1.47^a^
Propionic acid	3.11 ± 1.13^b^	3.74 ± 0.98^a^
Total organic acid content	158.4 ± 2.99^b^	183.53 ± 3.47^a^

FBDSM, fermented bean dregs and soybean meal; MEFC, mineral element fermentation complexes. Values expressed as mean ± standard deviation (n = 3).

Means with different letters are significantly different (P < 0.05).

### Effect of fermentation on Fe^2+^ and Fe^3+^ redox

**FIGURE 5:**
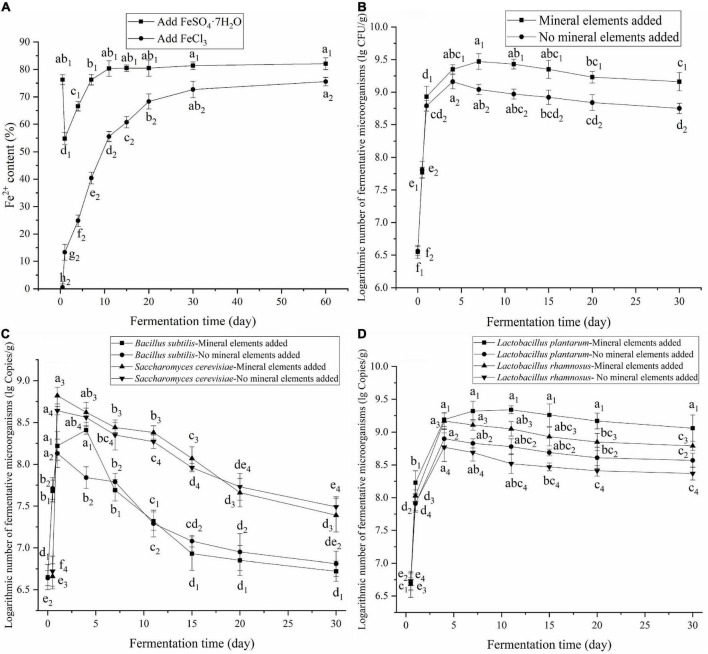
**(A)** Effect of fermentation on the redox of Fe^2+^ and Fe^3+^; **(B)** Effect of mineral elements on the number of viable microorganisms; **(C)** Effect of mineral elements on the growth of *Bacillus subtilis* and *Saccharomyces cerevisiae*; **(D)** Effect of mineral elements on the growth of *Lactobacillus*. Values expressed as mean ± standard deviation (*n* = 3).

Fe is important for laying hens and is directly related to hemoglobin synthesis and O_2_ transport and utilization (30). Fe^2+^ is easily oxidized to Fe^3+^, and Fe^3+^ must be transformed to Fe^2+^ before it can be used (31). FBDSM has good antioxidant properties that prevented the oxidation of Fe^2+^ to Fe^3+^ and promoted the reduction of Fe^3+^ to Fe^2+^. As shown in Figure 5A, the Fe^2+^ content of FBDSM with FeSO_4_⋅7H_2_O first decreased, then increased, and leveled off after 11 days. The Fe^2+^ content of FBDSM with FeCl_3_ continued to increase and then leveled off after 20 days. Because of the residual oxygen in the fermentation bag during the initial fermentation stage, Fe^2+^ was rapidly oxidized to Fe^3+^; after 1 day of fermentation, the Fe^2+^ content of FBDSM with FeSO_4_⋅7H_2_O was reduced to 54.84%. As fermentation progressed, oxygen was exhausted and reducibility increased; thus, Fe^3+^ was gradually reduced to Fe^2+^. On the 60th day of the two experiments, the Fe^2+^ contents were as high as 82.11 and 75.56%. Therefore, effective antioxidant protection against Fe^2+^ oxidation lasts at least 60 days. For fermentation complexation, Fe was added after aerobic fermentation to protect Fe^2+^.

### Effect of the mineral elements on fermenting microorganisms

Above a certain concentration range, mineral elements are toxic to microorganisms, and different microorganisms have distinct tolerances for mineral elements. As shown in Figure 5B, at the added concentrations, the mineral elements had a little toxic effects on the fermenting microorganisms, and the number of live fermenting microorganisms even increased. During early fermentation, the addition of mineral elements promoted the growth of *B. subtilis* and *S. cerevisiae*. The maximum numbers of *B. subtilis* and *S. cerevisiae* increased from 1.35 × 10^8^ to 2.57 × 10^8^ copies/g and from 4.37 × 10^8^ to 6.61 × 10^8^ copies/g, respectively (Figure 5C). The addition of mineral elements also promoted the growth of *L. plantarum* and *L. rhamnosus*. The maximum numbers of *L. plantarum* and *L. rhamnosus* increased from 7.94 × 10^8^ to 2.19 × 10^9^ and 5.89 × 10^8^ to 1.48 × 10^9^ copies/g, respectively (Figure 5D). Adding the minerals increased the concentrations of these elements that necessary for the growth and metabolism of the fermenting microorganisms and thus promoted their growth. The added CaCO_3_ increased protease activity, thus promoting the degradation of proteins into small peptides, which are more easily absorbed and utilized. CaCO_3_ also had a buffering effect against the produced organic acids and improved the low pH environment generated by the fermenting microorganisms. However, during late fermentation, the large amount of organic acids produced inhibited the growth of the fermenting microorganisms, leading to death, especially *B. subtilis* and *S. cerevisiae*.

### Effect of mineral element fermentation complexes on the production performance of laying hens

Compared with the control group, the average daily feed intake of laying hens fed MEFC was higher (Table 5). This may be because the MEFC contains organic acids, amino acids, and other components that gave the feed a strong acidic flavor, which stimulated feed intake. Feeding with MEFC also increased the egg production rate and average egg weight (Table 5), suggesting that MEFC improved the absorption of mineral elements and other nutrients. The broken soft egg rate and average eggshell thickness are closely related to the absorption of mineral elements, such as Ca. The broken soft egg rate of laying hens fed MEFC was lower than that of the control group, and the difference was significant from 31 to 60 days (6.18, 8.81%). The average eggshell thickness in the experimental group was higher than that in the control group; however, the difference was not statistically significant. These results indicated that MEFC could improve the absorption and utilization of mineral elements, especially Ca.

**TABLE 5:** Effect of mineral element fermentation complexes on production performance of laying hens.

Items	Experimental group, 1–30 days	Control group, 1–30 days	Experimental group, 31–60 days	Control group, 31–60 days
Average daily feed intake, g	100 ± 9.11^a^	87.66 ± 10.08^b^	98.2 ± 12.23^a^	88.95 ± 8.45^b^
Egg production rate, %	88.71 ± 1.56^a^	84.66 ± 2.35^b^	86.12 ± 1.15^a^	81.78 ± 2.11^b^
Average egg weight, g	55.89 ± 1.01^a^	51.17 ± 1.19^b^	56.2 ± 1.16^a^	52.78 ± 2.37^b^
Broken soft egg rate, %	8.43 ± 2	9.32 ± 1.62	6.18 ± 1.71^b^	8.81 ± 1.9^a^
Average eggshell thickness, mm	0.34 ± 0.03	0.31 ± 0.05	0.33 ± 0.03	0.30 ± 0.06

Means with different letters marked between two groups are significantly different (P < 0.05).

### Effect of mineral element fermentation complexes on the mineral element contents in the blood, liver, heart, and feces

As shown in Table 6, the Ca and Fe contents in the blood, liver, and heart; Zn content in the blood; and Cu content in the heart of the experimental group were significantly higher than those of the control group. However, there were no significant differences in the Cu and Mn contents in the blood; Cu, Mn, and Zn contents in the liver; and Mn and Zn contents in the heart between the two groups. The Ca, Fe, Cu, Mn, and Zn contents in the feces of laying hens fed MEFC were lower than those in the control group. The Cu and Mn contents from 31 to 60 days and the Ca and Fe contents were significantly different between the two groups. These results indicated that MEFC promoted the absorption of mineral elements and reduced the excretion of mineral elements in laying hens compared with inorganic mineral elements, which is consistent with the results of Meng et al. (32), who showed that supplementation with methionine manganese chelate improved tissue trace element deposition.

**TABLE 6:** Effect of MEFC on contents of mineral elements in blood, liver, heart and feces.

Items	Ca, mg/g	Fe, mg/g	Cu, mg/g	Mn, mg/g	Zn, mg/g
Blood of experimental group	0.63 ± 0.05^a^	2.16 ± 0.12^a^	0.059 ± 0.008	0.0015 ± 0.0004	0.035 ± 0.009^a^
Blood of control group	0.14 ± 0.02^b^	1.64 ± 0.09^b^	0.041 ± 0.009	0.0013 ± 0.0006	0.015 ± 0.003^b^
Liver of experimental group	0.41 ± 0.1^a^	0.53 ± 0.03^a^	0.095 ± 0.011	0.006 ± 0.001	0.13 ± 0.02
Liver of control group	0.06 ± 0.01^b^	0.27 ± 0.08^b^	0.089 ± 0.01	0.004 ± 0.001	0.12 ± 0.03
Heart of experimental group	0.22 ± 0.06^a^	0.61 ± 0.03^a^	0.087 ± 0.011^a^	0.014 ± 0.006	0.26 ± 0.04
Heart of control group	0.13 ± 0.03^b^	0.38 ± 0.08^b^	0.052 ± 0.008^b^	0.010 ± 0.004	0.24 ± 0.03
Feces of experimental group	19.99 ± 1.5^b^	0.38 ± 0.03^b^	0.055 ± 0.011^b^	0.18 ± 0.03^b^	0.15 ± 0.04
Feces of control group	26.8 ± 0.89^a^	0.71 ± 0.18^a^	0.074 ± 0.008^a^	0.25 ± 0.04^a^	0.22 ± 0.03

Means with different letters marked between two groups are significantly different (P < 0.05).

### Effect of mineral element fermentation complexes on the intestinal morphology of laying hens

Nutrients are mainly absorbed in the small intestine, and the higher the villus height to crypt depth ratio in the small intestine, the greater the intestinal absorption. Intestinal paraffin sections of laying hens are shown in Supplementary Figure 1. The villus height to crypt depth ratios were higher in the experimental group than in the control group (Table 7), and the differences were significant in the jejunum (14.32 vs. 11.46) and ileum (10.39 vs. 8.52). This may be because MEFC contains a large number of probiotics, which improved intestinal morphology.

**TABLE 7:** Effect of mineral element fermentation complexes on intestinal morphology of laying hens.

Items	Experimental group	Control group
Duodenal villus height (DVH), μm	1484.7 ± 77.11	1271.53 ± 86.54
Duodenal crypt depth (DCD), μm	141.97 ± 21.63	127.45 ± 18.75
DVH/DCD	10.46 ± 1.96	9.98 ± 1.1
Jejunal villus height (JVH), μm	992.19 ± 47.63	973.22 ± 36.56
Jejunal crypt depth (JCD), μm	69.28 ± 9.8	84.96 ± 6.97
JVH/JVC	14.32 ± 0.87^a^	11.46 ± 0.64^b^
Ileal villus height (IVH), μm	1066.35 ± 38.4	775.03 ± 40.84
Ileal crypt depth (ICD), μm	102.66 ± 7.75	90.95 ± 6.13
IVH/ICD	10.39 ± 1.04^a^	8.52 ± 0.72^b^

Means with different letters marked between two groups of VH/CD are significantly different (P < 0.05).

### Effect of mineral element fermentation complexes on blood routine indexes of laying hens

WBC are an important immune component and phagocytize invading bacteria. As shown in Table 8, the number of WBC was higher in the experimental group than in the control group; however, the difference was not significant, indicating that MEFC may enhance the immune function of laying hens. Kim et al. (33) found that probiotics composed of *B. subtilis*, *Lactobacillus*, and yeast significantly increased the number of WBC in the blood of laying hens. MEFC is rich in probiotics, which may be one of the reasons why the number of WBC was higher in the experimental group than in the control group. RBC, hemoglobin, and hematocrit are important indicators of anemia and iron nutritional status. RBC have the most abundant in animal blood and are the main media for the body to transport oxygen through the blood. Studies have shown that Fe and Cu are directly related to hemoglobin synthesis (34). As shown in Table 8, the number of RBC, hemoglobin levels, and the hematocrit of laying hens fed MEFC (3.43 × 10^12^/L, 176 g/L, and 43.3%, respectively) were significantly higher than those of the control group (2.22 × 10^12^/L, 135 g/L, and 30%, respectively), indicating that supplementation of Fe, Cu, and other minerals via MEFC increased RBC and hemoglobin significantly better than inorganic mineral salts. This may be related to the fact that the Fe in MEFC was mainly Fe^2+^. Fermentation of MEFC creates an anaerobic environment, which protects Fe^2+^ from oxygen thus avoiding oxidation. In addition, the fermentation itself has good redox properties. It can reduce the oxidation of Fe^3+^ to Fe^2+^ and provides antioxidant protection on Fe^2+^. Therefore, feeding laying hens MEFC can enhance oxygen transport and prevent anemia.

**TABLE 8:** Effect of MEFC on blood routine and biochemical indexes of laying hens.

Items	Experimental group	Control group
WBC, 10^^11^/L	2.42 ± 0.09	2.16 ± 0.13
RBC, 10^^12^/L	3.43 ± 0.26^a^	2.22 ± 0.17^b^
Hemoglobin, g/L	176 ± 5.3^a^	135 ± 4.9^b^
Hematocrit, %	43.3 ± 1.98^a^	30 ± 1.85^b^
ALT, U/L	2.2 ± 0.95	2.6 ± 1.3
AST, U/L	394.7 ± 16.64^b^	421 ± 18.31^a^
Urea, mmol/L	0.97 ± 0.15	0.91 ± 0.18
Uric acid, μmol/L	312.6 ± 15.67^b^	353.1 ± 17.35^a^
Blood glucose, mmol/L	14.02 ± 0.28	16.63 ± 0.32
TCHOL, mmol/L	5.85 ± 0.41^a^	2.07 ± 0.39^b^
HDL-C, mmol/L	1.54 ± 0.56^a^	0.59 ± 0.08^b^
LDL-C, mmol/L	2.74 ± 0.46^a^	0.69 ± 0.06^b^

WBC, white blood cells; RBC, red blood cells; ALT, alanine transaminase; AST, aspartate transaminase; TCHOL, total cholesterol; HDL-C, high-density cholesterol; LDL-C, low-density cholesterol. Means with different letters marked between two groups are significantly different (P < 0.05).

### Effect of mineral element fermentation complexes on biochemical indexes of laying hens

ALT and AST mainly exist in the cytoplasm and mitochondria of liver cells and are two important indexes of liver function. When the liver is damaged, cell membrane permeability and transaminase activity increase. The activity levels of ALT and AST were lower in the experimental group than in the control group; however, the difference in ALT between the two groups was not significant (Table 8), indicating that MEFC reduced the damage to the liver caused by mineral elements. Uric acid reflects protein metabolism in laying hens. The lower the uric acid content, the higher the protein metabolism and feed conversion rate. As shown in Table 8, the uric acid content in the experimental group (312.6 μmol/L) was lower than that in the control group (353.1 μmol/L), indicating that MEFC may increase protein metabolism. Mn can stimulate cholesterol synthesis, and Mn deficiency affects carbohydrate and lipid metabolism. The activity levels of enzymes related to lipid metabolism and glucose metabolism are regulated by Zn, and Zn supplementation can promote the metabolism and absorption of blood glucose and lipids. Abd El-Hack et al. (35) found that feeding zinc methionine chelate promoted lipid metabolism and absorption in laying hens. The blood glucose level was lower in the experimental group than in the control group, but the difference was not significant. TCHOL, HDL-C, and LDL-C levels were significantly higher in the experimental group than in the control group. These results indicated that MEFC promoted the absorption of Mn and Zn, as well as the metabolism and absorption of lipids and sugars.

## Conclusion

The optimized conditions for fermentation complexation process developed in this study were as follows: mineral elements (with 4% CaCO_3_) were added after aerobic fermentation, and 15% wheat flour middling were added to the medium followed by 34°C, 5% inoculum size, and 11 days of fermentation. Under these conditions, the complexation rates of Ca, Fe, Cu, Mn, and Zn were 90.62, 97.24, 73.33, 94.64, and 95.93%, respectively. The small peptide, amino acid, and organic acid contents were 41.62%, 48.09 mg/g, and 183.53 mg/g, respectively. The Fe in MEFC was mainly ferrous ions. The fermentation had a good antioxidant effect on ferrous ions, and the antioxidant protection period was at least 60 days. The added minerals promoted microbial growth, protein degradation, and organic acid secretion and significantly improved fermentation efficiency. The animal experiments showed that MEFC had positive effects on production performance, mineral absorption, intestinal morphology, and blood routine and biochemical indexes of laying hens. This study combines fermentation and mineral element complexation to obtain fermentation complexes containing multiple complexation types, which makes it possible to efficiently supplement minerals for laying hens at low cost and provides important guidance for the development of mineral element fermentation complexes for laying hens.

## Data availability statement

The original contributions presented in this study are included in the article/Supplementary material, further inquiries can be directed to the corresponding authors.

## Ethics statement

The animal study was reviewed and approved by the Institutional Animal Care and Use Committee of Jiangsu University.

## Author contributions

HC conceived and designed the experiments. XH performed the majority of experiments and wrote the manuscript. KL and ZW used the software. ZN, EG, YY, and XW supervised this study. All authors have read and approved the final manuscript.

## References

[1] Londero A Rosa AP Luiggi FG Fernandes MO Guterres A de Moura S Effect of supplementation with organic and inorganic minerals on the performance, egg and sperm quality and, hatching characteristics of laying breeder hens. *Anim Reprod Sci.* (2020) 215:106309. 10.1016/j.anireprosci.2020.106309 32216930

[2] Yang KL Hu SJ Mu R Qing YQ Xie L Zhou LY Effects of different patterns and sources of trace elements on laying performance, tissue mineral deposition, and fecal excretion in laying hens. *Animals.* (2021) 11:1164. 10.3390/ani11041164 33921551 PMC8072985

[3] Pereira CG Rabello CB Barros MR Manso H Santos M. Zinc, manganese and copper amino acid complexed in laying hens’ diets affect performance, blood parameters and reproductive organs development. *PLoS One.* (2020) 15:e0239229. 10.1371/journal.pone.0239229 33147220 PMC7641365

[4] Wang J Wang WW Qi G Cui CF Wu SG Zhang HJ Effects of dietary *Bacillus subtilis* supplementation and calcium levels on performance and eggshell quality of laying hens in the late phase of production. *Poult Sci.* (2021) 100:100970. 10.1016/j.psj.2020.12.067 33518333 PMC7936213

[5] Hunt JR. Bioavailability of iron, zinc, and other trace minerals from vegetarian diets. *Am J Clin Nutr.* (2003) 78(3 Suppl.):633S–9S. 10.1093/ajcn/78.3.633S 12936958

[6] Handa V Sharma D Kaur A Arya SK. Biotechnological applications of microbial phytase and phytic acid in food and feed industries. *Biocatal Agric Biotechnol.* (2020) 25:101600. 10.1016/j.bcab.2020.101600

[7] Bai SP Peng JL Zhang KY Ding XM Wang JP Zeng QF Effects of dietary iron concentration on manganese utilization in broilers fed with manganese-lysine chelate-supplemented diet. *Biol Trace Elem Res.* (2020) 194:514–24. 10.1007/s12011-019-01780-w 31933278

[8] Lyu B Wang H Swallah MS Fu HL Shen Y Guo ZW Structure, properties and potential bioactivities of high-purity insoluble fibre from soybean dregs (*Okara*). *Food Chem.* (2021) 364:130402. 10.1016/j.foodchem.2021.130402 34175627

[9] Wang RY Dong PS Zhu YY Yan MC Liu W Zhao YJ Bacterial community dynamics reveal its key bacterium, *Bacillus amyloliquefaciens* ZB, involved in soybean meal fermentation for efficient water-soluble protein production. *LWT.* (2021) 135:110068. 10.1016/j.lwt.2020.110068

[10] Heng XY Chen HY Lu CX Feng T Li KY Gao EB. Study on synergistic fermentation of bean dregs and soybean meal by multiple strains and proteases. *LWT.* (2022) 154:112626. 10.1016/j.lwt.2021.112626

[11] Hou H Wang SK Zhu X Li QQ Fan Y Cheng D A novel calcium-binding peptide from Antarctic krill protein hydrolysates and identification of binding sites of calcium-peptide complex. *Food Chem.* (2018) 243:389–95. 10.1016/j.foodchem.2017.09.152 29146354

[12] Standardization Administration of China. *Chinese National Standard GB 5009.268-2016.* Beijing: Standardization Administration of China (2016).

[13] Shi CY Zhang Y Lu ZQ Wang YZ. Solid-state fermentation of corn-soybean meal mixed feed with *Bacillus subtilis* and *Enterococcus faecium* for degrading antinutritional factors and enhancing nutritional value. *J Anim Sci Biotechnol.* (2017) 8:50. 10.1186/S40104-017-0184-2 28603613 PMC5465572

[14] Moghaddam TK Zhang J Du GC. UvrA expression of *Lactococcus lactis* NZ9000 improve multiple stresses tolerance and fermentation of lactic acid against salt stress. *J Food Sci Technol.* (2017) 54:639–49. 10.1007/s13197-017-2493-z 28298677 PMC5334222

[15] Stookey L. Ferrozine—A new spectrophotometric reagent for Iron. *Anal Chem.* (1970) 42:779–81. 10.1021/ac60289a016

[16] ISO. *ISO: 4833:2003.* Geneva: ISO (2003).

[17] Yu Z Dong B Lu W. Dynamics of bacterial community in solid-state fermented feed revealed by 16S rRNA. *Lett Appl Microbiol.* (2009) 49:166–72. 10.1111/j.1472-765X.2009.02636.x 19413759

[18] Standardization Administration of China. *Chinese Agriculture Industry Standard NY/T 2066-2011.* Beijing: Standardization Administration of China (2011).

[19] Costa GN Vilas-Bôas GT Vilas-Boas LA Miglioranza LHS. In silico phylogenetic analysis of lactic acid bacteria and new primer set for identification of *Lactobacillus plantarum* in food samples. *Eur Food Res Technol.* (2011) 233:233–41. 10.1007/s00217-011-1508-7

[20] NRC. *Nutrient Requirements of Poultry (9th Rev. Ed.)*. Washington, DC: National Acadmic Press (1994).

[21] Wang M Wang LX Tan X Wang L Xiong X Wang YC The developmental changes in intestinal epithelial cell proliferation, differentiation, and shedding in weaning piglets. *Anim Nutr.* (2022) 9:214–22. 10.1016/j.aninu.2021.11.006 35600553 PMC9092860

[22] Sun XY Zhao Y Liu LL Jia B Zhao F Huang WD Copper tolerance and biosorption of *Saccharomyces cerevisiae* during alcoholic fermentation. *PLoS One.* (2015) 10:e0128611. 10.1371/journal.pone.0128611 26030864 PMC4452488

[23] Fan Y Tian LL Xue Y Li ZJ Hou H Xue CH. Characterization of protease and effects of temperature and salinity on the biochemical changes during fermentation of Antarctic krill. *J Sci Food Agric.* (2017) 97:3546–51. 10.1002/jsfa.8209 28078684

[24] Li JY Zhang LW Han X Yi HX Guo CF Zhang YC Effect of incubation conditions and possible intestinal nutrients on cis-9, trans-11 conjugated linoleic acid production by *Lactobacillus acidophilus* F0221. *Int Dairy J.* (2013) 29:93–8. 10.1016/j.idairyj.2012.10.013

[25] Shue G Yang H Chen H Zhang Q Tian Y. Effect of incubation time, inoculum size, temperature, pasteurization time, goat milk powder and whey powder on ACE inhibitory activity in fermented milk by *L. plantarum* LP69. *Acta Sci Pol Technol Aliment.* (2015) 14:107–16. 10.17306/J.AFS.2015.2.12 28068008

[26] Feng R Chen L Chen KP. Fermentation trip: amazing microbes, amazing metabolisms. *Ann Microbiol.* (2018) 68:717–29. 10.1007/s13213-018-1384-5

[27] Sun X Sarteshnizi RA Boachie RT Okagu OD Abioye RO Neves RP Peptide-mineral complexes: understanding their chemical interactions, bioavailability, and potential application in mitigating micronutrient deficiency. *Foods.* (2020) 9:1–17. 10.3390/foods9101402 33023157 PMC7601898

[28] Zheng L Liu MQ Sun JD Wu B He BF. Sodium ions activated phosphofructokinase leading to enhanced d-lactic acid production by *Sporolactobacillus inulinus* using sodium hydroxide as a neutralizing agent. *Appl Microbiol Biotechnol.* (2017) 101:3677–87. 10.1007/s00253-017-8120-0 28190098

[29] Hui S Ghergurovich JM Morscher RJ Jang C Teng X Lu WY Glucose feeds the TCA cycle via circulating lactate. *Nature.* (2017) 551:115–8. 10.1038/nature24057 29045397 PMC5898814

[30] Wang CY Babitt JL. Liver iron sensing and body iron homeostasis. *Blood.* (2019) 133:18–29. 10.1182/blood-2018-06-815894 30401708 PMC6318427

[31] O’Loughlin IB Kelly PM Murray BA FitzGerald RJ Brodkorb A. Molecular characterization of whey protein hydrolysate fractions with ferrous chelating and enhanced iron solubility capabilities. *J Agric Food Chem.* (2015) 63:2708–14. 10.1021/jf505817a 25716093

[32] Meng T Gao L Xie C Xiang Y Huang Y Zhang Y Manganese methionine hydroxy analog chelated affects growth performance, trace element deposition and expression of related transporters of broilers. *Anim Nutr.* (2021) 7:481–7. 10.1016/j.aninu.2020.09.005 34258436 PMC8245798

[33] Kim CH Woo KC Kim GB Park YH Paik IK. Effects of supplementary multiple probiotics or single probiotics on the performance, intestinal microflora, immune response of laying hens and broilers. *Korean J Poult Sci.* (2010) 37:211–7. 10.5536/KJPS.2010.37.1.051 25797497

[34] Eisenstein RS. Iron regulatory proteins and the molecular control of mammalian iron metabolism. *Annu Rev Nutr.* (2000) 20:627–62. 10.1146/annurev.nutr.20.1.627 10940348

[35] Abd El-Hack ME Alagawany M Amer SA Arif M Wahdan KMM El-Kholy MS. Effect of dietary supplementation of organic zinc on laying performance, egg quality and some biochemical parameters of laying hens. *J Anim Physiol Anim Nutr.* (2018) 102:e542–9. 10.1111/jpn.12793 28990706

