# Supplementary material for: Complexation of multiple mineral elements by fermentation and its application in laying hens
Source: Front Nutr. 2022 Sep 29;9:1001412. doi: 10.3389/fnut.2022.1001412 (PMC9556719; doi:10.3389/fnut.2022.1001412)
Supplement: Supplementary file 1 [file Table_1.DOCX]

**Supplementary Material**

**Supplementary Table 1**

Contents of small peptide and organic acid in FBDSM under initial fermentation process

| Items | FBDSM with no mineral elements |
| --- | --- |
| Small peptide content, % | 24.59 ± 0.93 |
| Organic acid content, mg/g | 120.63 mg/g |

FBDSM = fermented bean dregs and soybean meal.

Values expressed as mean ± standard deviation (n = 3).

**Supplementary Table 2**

Small peptide contents of FBDSM and MEFC after fermentation complexation

| Item | FBDSM | MEFC |
| --- | --- | --- |
| Small peptide content, % | 39.56 ± 1.15 | 41.62 ± 0.78 |

FBDSM = fermented bean dregs and soybean meal; MEFC = mineral element fermentation complexes.

Values expressed as mean ± standard deviation (n = 3).

Means with different letters are significantly different (*P* < 0.05).


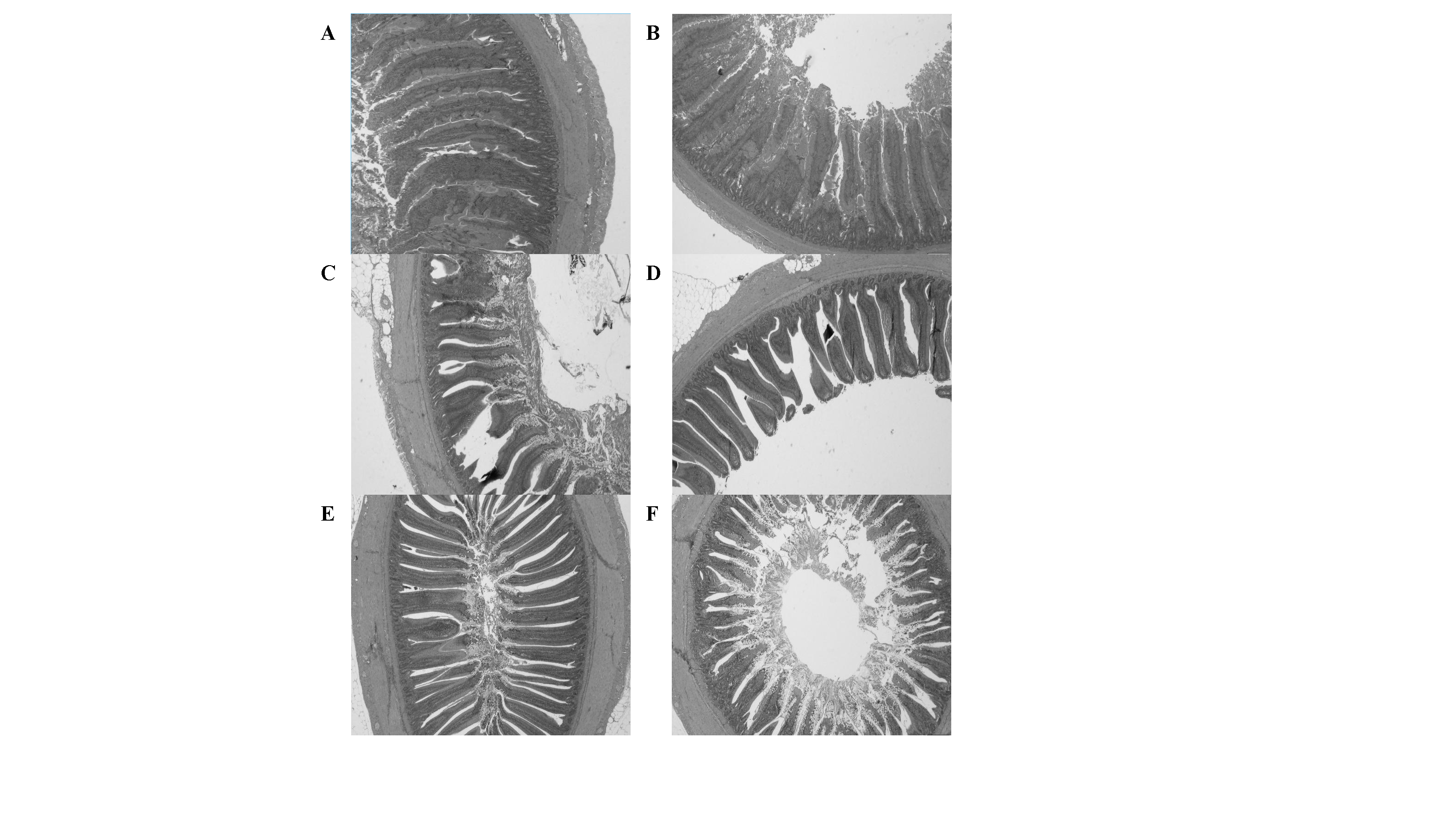


**Supplementary Figure 1.** **(A)** Paraffin section of duodenum in the experimental group; **(B)** Paraffin section of duodenum in the control group; **(C)** Paraffin section of jejunum in the experimental group; **(D)** Paraffin section of jejunum in the control group; **(E)** Paraffin section of ileum in the experimental group; **(F)** Paraffin section of ileum in the control group
